# Real-Time Alpine Measurement System Using Wireless Sensor Networks

**DOI:** 10.3390/s17112583

**Published:** 2017-11-09

**Authors:** Sami A. Malek, Francesco Avanzi, Keoma Brun-Laguna, Tessa Maurer, Carlos A. Oroza, Peter C. Hartsough, Thomas Watteyne, Steven D. Glaser

**Affiliations:** 1 Department of Civil and Environmental Engineering, University of California, Berkeley, CA 94720, USA; avanzi.f@berkeley.edu (F.A.); tmaurer13@berkeley.edu (T.M.); coroza@berkeley.edu (C.A.O.); glaser@berkeley.edu (S.D.G.); 2 French Institute for Research in Computer Science and Automation (Inria), 2 Rue Simone IFF, 75012 Paris, France; keoma.brun-laguna@inria.fr (K.B.-L.); thomas.watteyne@inria.fr (T.W.); 3 Department of Land, Air, and Water Resources, University of California, Davis, CA 95616, USA; phartsough@ucdavis.edu

**Keywords:** wireless sensor networks, ground measurement system, mountain hydrology, snow pack, internet of things, real-time monitoring system

## Abstract

Monitoring the snow pack is crucial for many stakeholders, whether for hydro-power optimization, water management or flood control. Traditional forecasting relies on regression methods, which often results in snow melt runoff predictions of low accuracy in non-average years. Existing ground-based real-time measurement systems do not cover enough physiographic variability and are mostly installed at low elevations. We present the hardware and software design of a state-of-the-art distributed Wireless Sensor Network (WSN)-based autonomous measurement system with real-time remote data transmission that gathers data of snow depth, air temperature, air relative humidity, soil moisture, soil temperature, and solar radiation in physiographically representative locations. Elevation, aspect, slope and vegetation are used to select network locations, and distribute sensors throughout a given network location, since they govern snow pack variability at various scales. Three WSNs were installed in the Sierra Nevada of Northern California throughout the North Fork of the Feather River, upstream of the Oroville dam and multiple powerhouses along the river. The WSNs gathered hydrologic variables and network health statistics throughout the 2017 water year, one of northern Sierra’s wettest years on record. These networks leverage an ultra-low-power wireless technology to interconnect their components and offer recovery features, resilience to data loss due to weather and wildlife disturbances and real-time topological visualizations of the network health. Data show considerable spatial variability of snow depth, even within a 1 km${}^{2}$ network location. Combined with existing systems, these WSNs can better detect precipitation timing and phase in, monitor sub-daily dynamics of infiltration and surface runoff during precipitation or snow melt, and inform hydro power managers about actual ablation and end-of-season date across the landscape.

## 1. Introduction

Snow represents the predominant winter land surface cover for 50% of North America, Europe and Asia [1]. Together with glaciers, snow provides one-sixth of the world’s population with fresh water [2]. As a result, economic valuations of snow resources run in the trillions of dollars [3]. Runoff from snowmelt is also an important source of hydropower for populous regions such as the Himalayas in Asia [4], the Sierra Nevada and Rocky Mountains in the Western United States [5,6], and the Alps in Europe [7]. As climate change and population growth increase strain on water and energy systems, it is crucial to improve monitoring of the snowpack and snowmelt processes in the world’s mountain regions to enhance control and forecasting of water supplies [8].

Traditional methods of forecasting snowmelt and runoff rely on regressing current conditions against historical data. These approaches become unreliable in the presence of climate change, especially with the predicted increased frequency of extreme weather events [9]. More robust alternatives consist of physically-based models such as the Precipitation Runoff Modeling System (PRMS) [10], ALPINE3D [11], or Snowmelt Runoff Model (SRM) [12]. They provide a more realistic representation of water-budget fluxes that ultimately translates into an adaptive decision support system for reservoir management [5], water conservation, hydro-power optimization and flood control. These models require a large amount of input data as well as a spatially explicit characterization of basin properties and parameters [13]. One of the main challenges is therefore to increase the spatial and temporal resolution of existing monitoring networks to provide the data necessary to improve the accuracy of snowmelt runoff forecasts.

Current snow measurement systems include: (i) manual ground-based snow courses [14], which measure single snapshots of snow properties in time at various scales (plot, transect, slope etc.); (ii) automated ground-based systems at point scale, such as snow pillows and snow depth sensors [15,16,17,18]; and (iii) terrestrial and airborne remote sensing products like laser scanning [19,20,21], Unmanned Aerial Vehicles [22,23,24], or satellite platforms [25]. While automatic ground systems provide data with a relatively high temporal resolution at specific, usually flat and open, locations, remote sensing measures snow patterns at larger geographic scales but with coarser temporal and spatial resolutions. Bridging the gap between these two sources of data fulfills the need for both spatially and temporally high-resolution information that current products cannot provide.

This article presents a recent deployment of a wireless sensor network (WSN) designed for snow hydrology, a new method of environmental sensing which collects and sends out data every 15 min from sensor node clusters covering tens of hectares. The location of each node corresponds to specific physiographic features known to impact snow distribution like elevation, aspect, slope, and canopy. These WSNs, deployed in the Feather River basin in the California Sierra Nevada (USA), constitutes the second generation of fully wireless systems deployed to the Sierra Nevada and aims primarily at assisting hydropower operations and ultimately water resources management at the State level. The design of first-generation WSNs [8] has been renovated to specifically fulfill hydropower real-time forecast needs in snow-dominated contexts. The deployment of these networks is the result of a partnership between the University of California, Pacific Gas & Electric, California Department of Water Resources, California Energy Commission, and the Institut de recherche en informatique et en automatique.

The remainder of this article is organized as follows. Section 2 describes the hardware and software design of the deployment, with a focus on how it addresses the resiliency of the system in harsh alpine conditions. Section 3 presents two types of results; Hydrologic results include the data collected during one of Northern California’s wettest water years (WY), 2017 and Networking results show the performance of the WSNs during this challenging WY, based on self-reported network health data. Section 4 discusses how this network compares to other wireless sensor networks and traditional snow survey techniques.

## 2. Materials and Methods

This section describes all the hardware (Section 2.1) and software (Section 2.3) in sufficient detail to allow the interested reader to replicate the results. This is greatly simplified as all the software is published under a BSD open-source license (https://github.com/realms-team/). Section 2.2 provides necessary information for understanding the low-power wireless networking technology we are using. Section 2.4 describes the methodology we employ when deploying a new network, further allowing the interested reader to replicate the results.

### 2.1. Hardware

**Figure 1:**
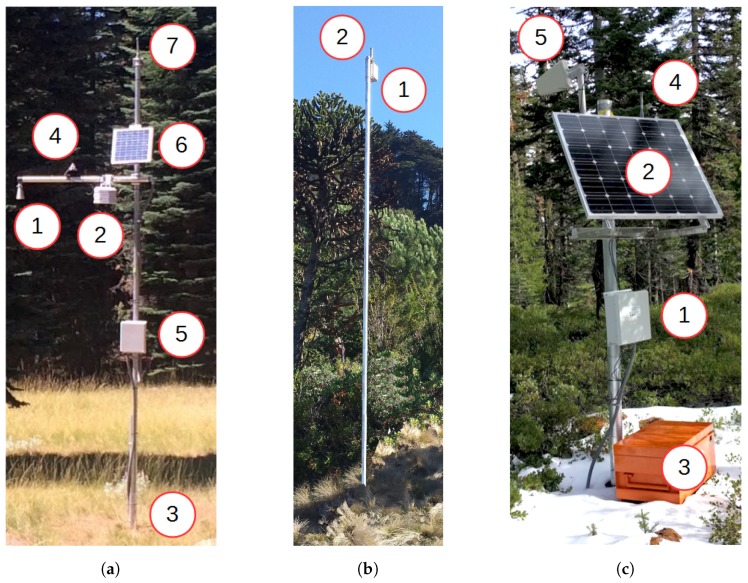
The hardware used. Circled numbers refer to specific modules of the system in Section 2.1. (**a**) Sensor station (Section 2.1.1); (**b**) Repeater node (Section 2.1.2); and (**c**) Base station (Section 2.1.3).

Four different types of hardware are used in the system:

- Sensor stations (Figure 1a) are installed at physiographically representative locations within network clusters and measures snow and meteorological variables, which are transmitted to the base station.
- In case the sensor station is too far from the base station for direct communication, repeater nodes (Figure 1b) are installed to serve as data relays. They also maintain the redundancy of a full mesh network.
- The base station (Figure 1c) serves as a collection point for all the data gathered by the sensor stations, and forwards this data to the server over a cellular Internet link.
- The server receives, stores and displays the data (not shown).

Each type of hardware is detailed in a subsequent section. Circled numbers (e.g., ①) refer to annotations in Figure 1.

#### 2.1.1. Sensor Station

A sensor station (Figure 1a) consists of a 5 m high schedule 80 aluminum pole with sensors attached, and a mote to control the sensors, make local calculations and communicate the sensor measurements to the base station. Up to 4 types of sensors can be mounted onto the pole.

- MB7363 Maxbotix ultrasonic range-finder ① can be mounted on the tip of the crossarm, oriented downwards. It measures the distance to ground or snow by measuring the round-trip time of an ultrasonic pulse. It has a resolution of 1 mm, an accuracy of 1%, and a range of 50 cm to 10 m. Like all ultrasonic devices, it is less accurate while it is snowing. We obtain the snow height by subtracting the distance measured when there is no snow.
- Temperature and relative humidity is measured by a Sensirion SHT25 sensor ②. It is enclosed in a radiation shield, and mounted about halfway across the crossarm.
- Decagon GS3 soil-moisture sensor ③ which measures soil dielectric constant, electric conductivity and temperature. Soil moisture is more accurately estimated via a calibration relation (http://manuals.decagon.com/Manuals/13822_GS3_Web.pdf). Results reported have not been locally calibrated. Two such sensors are installed per sensor station, at depths of 25 cm and 50 cm into the ground.
- Hukesflux LP02 pyranometer solar radiation sensor ④. One solar radiation sensor per WSN is installed in an open area. Unshaded solar radiation tends to be uniform across a 1–2 km${}^{2}$ area.

A sensor station typically features one ultrasonic range-finder, one temperature and relative humidity sensor, and two soil-moisture sensors (installed at different depths). In addition, one sensor station in the deployment features a solar radiation sensor.

These sensors are connected through wires to a NeoMote. The NeoMote by Metronome Systems (http://www.metronomesystems.com/) is a multi-purpose, ultra-low mote. It features a 16-bit ARM programmable system on chip (PSOC), a versatile micro-controller capable to interfacing to virtually any sensor and actuator. Up to 40 different types of sensors can be interfaced to the PSOC. It also features a SmartMesh IP wireless module by Analog Devices, allowing it to transfer its sensor measurements to the base station in a reliable, ultra low-power and secure way. Additionally, the NeoMote contains an SD card for local backup, and a real-time clock (RTC) for timestamping. The NeoMote and sensor are powered by a 17 Ah Li-Ion battery, which is recharged by a solar panel ⑥. A 2.4 GHz, 4 dBi, omnidirectional antenna is mounted on the top of the pole ⑦ to allow the wireless module to communicate. To install a sensor station, a steel U-channel is concreted into the ground. The main pole is then bolted onto the U-channel. This setup allows the pole to be removed when needed. All of the electronics are housed in a waterproof NEMA 4 fiberglass enclosure ⑤ attached to the pole. All sensor and solar panel wires are routed to the electronics box via the crossarm and metal-reinforced conduit to prevent exposure to weather and wildlife. They enter the box through conduit holes at the bottom of the box to prevent water from leaking into the enclosure. The cross-arm is bolted onto the main pole 4 m from the ground.

#### 2.1.2. Repeater Node

The role of the repeater node (Figure 1b) is to provide connectivity between the sensor stations and the base station and maintain the redundancy of the mesh network. Mechanically, it resembles the sensor station: it consists of a 5 m aluminum pole bolted to a U-channel. It is, however, much simpler than a sensor station, as it only contains a waterproof fiberglass enclosure ① with a Metronome Systems Wireless Sensing Relay Board. This node contains only the SmartMesh IP wireless module and is powered by a 17 Ah primary battery. An antenna is mounted on the top of the box ②.

#### 2.1.3. Base Station

The role of the base station (Figure 1c) is four-fold: (i) control and maintain the network, up to 100 nodes; (ii) collect the sensor measurements from the sensor stations; (iii) locally store the data; and (iv) transmit the data to the server on the Internet through a cellular connection.

**Figure 2:**
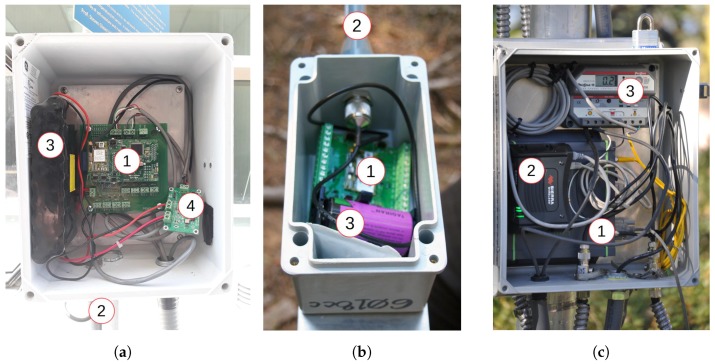
Contents of the electronics boxes: (**a**) Sensor station; (**b**) Repeater node; and (**c**) Base station.

**Figure 3:**
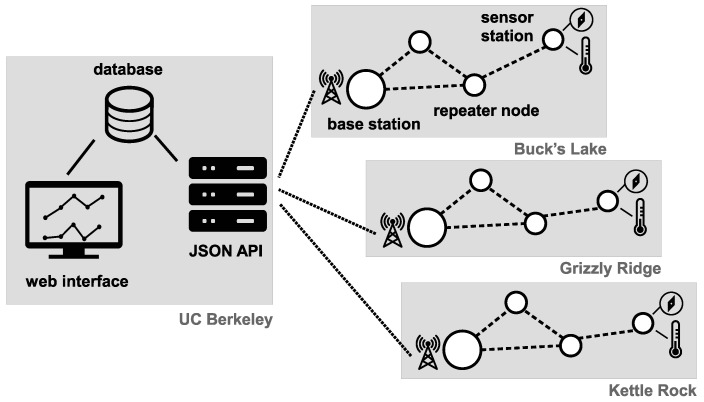
Software architecture (adapted from [28]).

**Figure 4:**
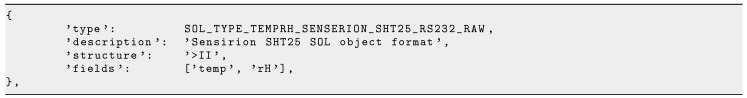
Excerpt of the SOL registry.

**Figure 5:**
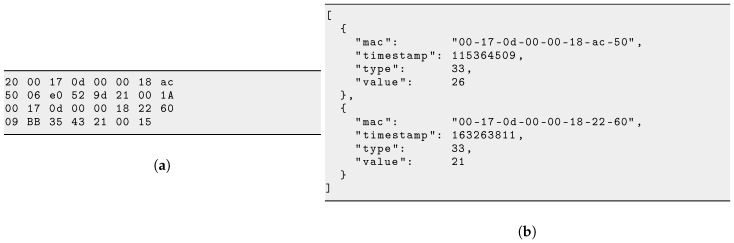
Different encodings of the same example compound SOL object (reproduced from [28]). (**a**)binary encoding (*31 bytes*); and (**b**) JSON encoding (*156 bytes*).

The base station is built around a 100 mm diameter aluminum pole. The waterproof fiberglass enclosure mounted 1.5 m from the ground contains several elements. First, it contains a Network Manager from Metronome Systems (Figure 2c, ①), which acts as the gateway of the SmartMesh IP network. The Manager contains two main elements: a SmartMesh IP module and a GNU/Linux computer consuming only 50 mA. They are connected to one another by an internal serial interface. This Network Manager uses a 2.4 GHz omni-directional antenna (Figure 1c, ④) to build a multi-hop mesh network with the sensor stations and repeater nodes. Second, it contains a Sierra Wireless AirLink GX450 cellular modem (Figure 2c, ②) for the Manager to connect to the Internet. This modem uses a directional antenna (Figure 1c, ⑤) which is pointed at the nearest cell phone tower. Manager and cellular modem are connected over Ethernet. Combined, the electronics consume around 200 mA. A 150 W solar panel (Figure 1c, ②) is used to charge two deep-cycle 66 Ah rechargeable sealed batteries which are enclosed in a Rigid box (Figure 1c, ③).

#### 2.1.4. Server

The role of the server (Figure 1c) is three-fold: (i) receive the data sent by the base stations of multiple deployments; (ii) store the data in a database; and (iii) offer a web interface to navigate and download the data. The server is rack-mounted and located at UC Berkeley. It is equipped with an 3 GHz Intel Core i7 CPU, a 1 TB drive, and 8 GB of RAM memory.

### 2.2. Low-Power Wireless Mesh Network

Each device (sensor station, repeater node, and base station) is equipped with the same SmartMesh IP LTC5902-IPM (http://www.linear.com/product/LTP5902-IPM SmartMesh IP Wireless 802.15.4e PCBA Module with Antenna Connector) wireless module. This module contains a combined radio and micro-controller system-on-chip, an antenna connector, and all required passives and crystals. It comes preprogrammed and takes care of all the networking aspects. It either runs entirely standalone (“master” mode) or can be driven by an external micro-controller through a serial interface (“slave” mode). When switched on, all of these devices form a low-power wireless multi-hop mesh network. The fact that it is multi-hop means that if a sensor station is too far from the base station to transmit its data directly, other device(s) serve as relays. Both sensor stations and repeater nodes can relay data; they are equivalent from a networking point of view. The fact that the network is a mesh means that a device connects with multiple other devices, providing redundancy, which leads to high end-to-end reliability. A device can make an arbitrary number of connections, meaning that, theoretically, the mesh could have hundreds of devices and excessive redundancy. However, to balance trade-offs like cost, logistics, and limiting the network’s footprint, we aimed to provide each node with at least two parent nodes to prevent single path failures. More information is available in Section 2.4. Figure 7 shows snapshots of the mesh network topology on three deployments (detailed description in Section 3.1).

In a SmartMesh IP network, all devices are tightly synchronized, with a maximum device-to-device de-synchronization below 15 μs across the network. Time is sliced up into slots; all communication in the network is orchestrated by a communication schedule. The schedule indicates to each node what to do in each time slot: transmit, listen or sleep. This allows the network to avoid internal interferences and keeps the energy consumption down. The network continuously optimizes the network, automatically making adjustments to the schedule when needed (e.g., a new device needs to publish more data, a wireless link breaks, etc.). A SmartMesh IP network is expected to yield over 99.999% end-to-end reliability. With an average current consumption below 50 μA, a device operates for over a decade when powered by a pair of AA batteries. Examples of applications of the time synchronization used by a SmartMesh IP network are detailed in [26,27]. The NIST-certified security provides confidentiality, integrity, and authentication to the communication.

SmartMesh IP is a proven technology; we chose to use it because our system operates well within the limits of a SmartMesh IP network. A SmartMesh IP network as a whole cannot generate more than 36 packets per second, with each packet carrying at most 90 bytes of application payload. In our case, each sensor station generates a 20-byte data packet every 15 min, well within the limit. The typically latency from a sensor station to the base station is in the order of 1-2 seconds. Given the relatively slow-moving nature of the data that we are measuring, this delay is perfectly acceptable.

### 2.3. Software Architecture

The Sensor Object Library (SOL) software architecture was fully developed for the deployments described in this article. The details about SOL System have been previously published in [28]. This section provides the necessary overview, illustrated by Figure 3.

SOL resides in four different locations: the sensor station (Section 2.3.2), the repeater node (Section 2.3.3), the base station (Section 2.3.4) and the server (Section 2.3.5). All the code developed is provided under a BSD open-source license (as an online addition to this paper, all source code can be found at https://github.com/realms-team). The code is being developed using state-of-the-art software development project management tools, and is production-ready.

#### 2.3.1. Sensor Object Library (SOL)

The driving concept in the design of SOL is that each sensor measurement or network statistics is represented as an atomic SOL object. Conceptually, this self-contained SOL object is formed by the fields listed in Table 1. This format is an equivalent to the well-known Type-Length-Value (TLV) scheme, to which we have added address and timestamp fields.

A publicly-maintained SOL registry (https://github.com/realms-team/sol/blob/master/registry.md) lists the different SOL object types, and for each the format of the value **V**. Figure 4 shows an excerpt of the SOL registry, on the format of a SOL object corresponding to the Sensirion SHT25 temperature and humidity sensor. It indicates that the value **V** field is 4 bytes long: a 2 byte temperature value followed by a 2 byte relative humidity value. The SOL registry currently contains 55 entries.

A SOL object can be encoded in 2 different formats, an example of which is shown in Figure 5. When the sensor station generates a SOL object, it encodes it in a compact binary format, typically 10–50 bytes. This is what the sensor station writes into the packets it sends to the base station across the low-power wireless mesh network. The base station converts the binary encoding into the equivalent JSON encoding. JSON [29] is a text-based encoding ubiquitous in machine-to-machine communication on the Internet, and well-supported by tools, including the database and web interface on the server.

We have developed Python and C core libraries to manipulate SOL objects, including serialization, de-serialization, conversion and validation routines. The software running on the sensor station, base station and server builds around these libraries.

#### 2.3.2. Sensor Station Firmware

The SmartMesh IP module of the sensor stations comes pre-programmed. The only modification applied is that it is configured to operate in “slave” mode, allowing the PSOC to drive it over a serial port. It is also configured to automatically join the network. The PSOC firmware handles the following basic tasks: (i) it samples the different external sensors; (ii) it saves those measurements locally on the SD card; and (iii) it sends that information to the SmartMesh IP module. These different steps are executed every 15 min; between those activity periods, the PSOC is in ultra low-power mode. The PSOC also implements advanced features, such as the ability to request the sensor station to resend some of the previous measurements stored on its SD card. The firmware comes with a library of drivers for the different sensors. Reading a sensor can be as simple as reading a single value over a digital bus (this is the case for example for the Sensirion temperature/humidity sensors). More advanced drivers include the one of the ultrasonic range finder, which triggers 28 snow depths measurements and reports their filtered average and standard deviation.

#### 2.3.3. Repeater Node Configuration

The repeater node’s SmartMesh IP module comes pre-programmed as well. We configure it so it runs in “master” mode: it joins and participates in the network without needing to be driven by an external micro-controller.

#### 2.3.4. Base Station Software

The SmartMesh IP module of the base station comes pre-programmed as manager for up to 100 nodes, and is used as-is. The GNU/Linux computer, running a Debian Jessie instance, handles the following tasks: (i) it drives the cellular modem connected to it; (ii) it waits for notifications from the SmartMesh IP manager, containing the sensor measurements generated by the sensor stations; (iii) it stores those notifications locally in a back-up file; (iv) it converts the SOL objects contained in the notifications from their binary to their JSON encoding; and (v) it sends these objects to the server. The cellular modem is configured to switch to low power standby mode when its input voltage drops below a threshold of 11V.

#### 2.3.5. Server Software

**Figure 6:**
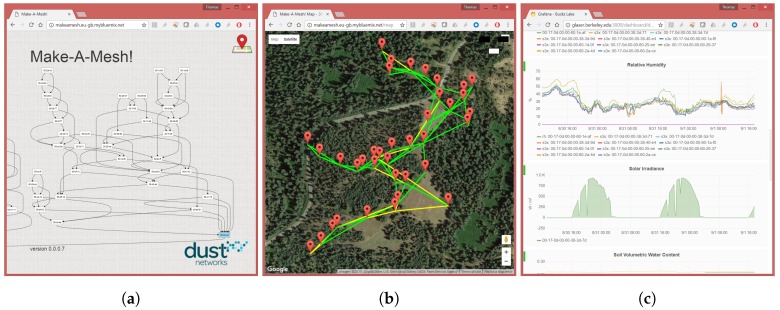
Web frontend (Bucks Lake deployment): (**a**) Topology view; (**b**) Map view; and (**c**) Data view.

The server runs Ubuntu 14.04.1 LTS, a flavor of GNU/Linux. Three base services are deployed: (i) a Python-based program offers a RESTful HTTPS/JSON interface for base stations to send their data to; (ii) an InfluxDB time series database (https://www.influxdata.com/) holds all the SOL objects; and (iii) a Grafana web frontend (https://grafana.com/) allows a user to navigate the data. The server builds a web frontend on top of these base services, allowing the user to see the logical topology of the network (Figure 6a), see the map of the network (Figure 6b), and navigate the sensor data (Figure 6c).

### 2.4. Deployment Strategy

The deployment strategy can be subdivided into four components: site selection, base station siting, sensor station placement, and repeater placement.

Site selection: At the highest level, the goal is to identify one or multiple deployment sites. A deployment site is roughly 1 km${}^{2}$. Our site selection process over the Feather River was driven by a desire to expand current monitoring capabilities on the North Fork, where most of the powerhouses are located, with particular focus on the under-monitored and largely undeveloped East Branch. Networks were chosen to be co-located with existing snow pillows that measure snow water equivalent (SWE). These sites where chosen by our partners Pacific Gas & Electric. Co-located SWE measurements enable better estimation of snow-water storage across the landscape. Sites were chosen to sample along a large elevation gradient, as hydrological processes in mountainous regions are driven by factors that change with elevation. Finally, sites were selected to capture hydrologic variability induced from a ridge that produces a rain shadow between the North Fork and the East Branch of the Feather River.

Base station siting: Once a deployment site is identified, the next step is to survey the 1 km${}^{2}$ area and identify where there is cellular connectivity, if any. Field teams survey the proposed network sites with a cellular modem attached to a directional antenna. The base station is placed where there is high cellular connectivity, as close as possible to the center of the field site. This minimizes the number of hops to the farthest nodes in the final mesh network, thereby reducing power consumption and increasing reliability. If no cellular connectivity is found, the back-up option is to use a satellite connection.

Sensor station placement: Once the possible locations for the base station are identified, the next step is to identify the locations of the sensor stations. This is done based on a combination of hydrologic and network considerations. The goal is to identify 12 locations (in the deployments covered in this article, the limit of number of sensor stations per deployment site is 12, based on budgetary considerations) within the 1 km${}^{2}$ deployment area which capture the variability of variables known to affect snow cover: slope, aspect, vegetation and elevation. This is done by a machine-learning program developed in our laboratory [30]. An additional constraint is that, given multiple potential sensor station locations, we prefer locations that are close to the base station to limit the number of repeaters. One node per site was installed at the same location of the snow pillow to enable direct comparisons between our measurements of snow depth and pillow SWE (henceforth, this node is referred to as the pillow node). At the same location, a rain gauge is usually available.

Repeater placement: Once the position of the sensor stations and the base station is determined, repeaters are added to connect the sensor stations to the base station and establish the network mesh. Prior studies have evaluated strategies for pre-computing optimal repeater placements for wireless mesh networks [31,32,33]. These methods often rely on simplifying assumptions, such as a flat environment and a fixed transmission range. Such assumptions are too restrictive for wireless-mesh networks in mountain environments, which feature terrain variability, complex spatial patterns of canopy cover, and variable snow depth, all of which affect path quality [34,35,36] and cause complicated multi-path effects. In practice, networks must be structured by field teams on the ground using real-time measurements of network health measured at the base station. The base station is placed near the center of the network, so members of the field team start there and build the network out towards each sensor station.

Three priorities guide the field teams’ selection of repeater placement: First, placements with an unobstructed path (i.e., free from terrain intersection or canopy cover) are prioritized over paths with obstructions. Second, field teams aim to ensure that the failure of a single node in the mesh cannot disconnect the network (i.e., that the final mesh be 2-vertex connected). This is not always possible if there is a limited budget for repeater placements. Where possible, 1-vertex-connected components of the graph are limited to nodes that are farthest away from the base station, so failure of the node will only affect a single sensor station. Third, a rule provided by Analog Devices, the manufacturer of the SmartMesh IP solution, requires that each node in the mesh must have at least 3 good neighbors [37]. A link between two devices is “good” when the quality is above 50%, i.e., over 50% of the packets exchanged between the neighbors are done so without retries. After the deployment, field teams evaluate the network statistics generated by the network and potentially add repeaters for sparsely connected regions.

## 3. Results

This section details three classes of results. Section 3.1 starts by detailing the deployments using the technology described in Section 2. Section 3.2 then details the hydrological information provided from the sensor measurements taken. Section 3.3 presents the networking results, i.e., it analyzes the performance of the low-power wireless mesh network.

### 3.1. Deployments

**Figure 7:**
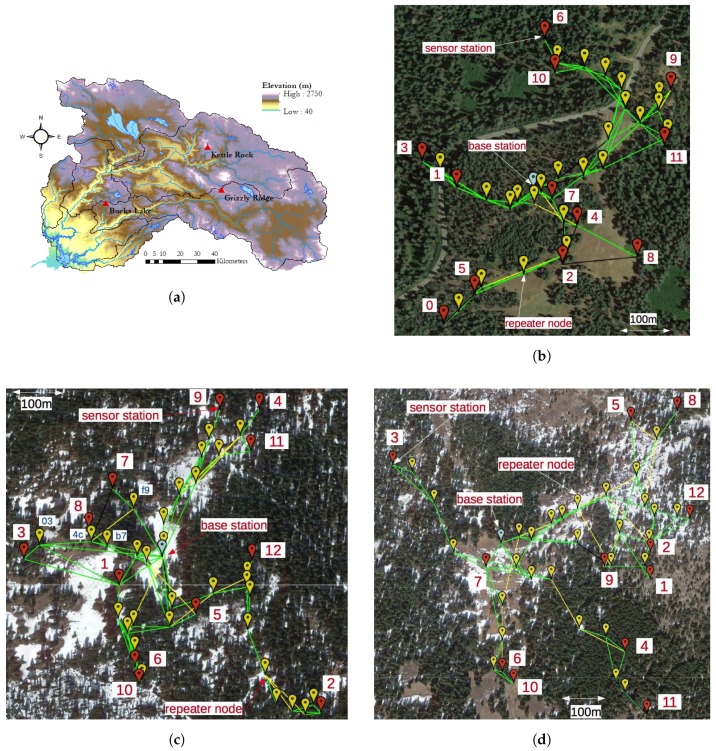
Maps of the deployments: (**a**) Elevation map of the Feather River basin, with the deployment locations indicated; (**b**) Bucks Lake deployment; (**c**) Grizzly Ridge deployment; and (**d**) Kettle Rock deployment.

The technology described in Section 2 has been deployed in three DWR-maintained independent sites across the Feather River basin in California, USA. The sites are Bucks Lake (BKL), Grizzly Ridge (GRZ) and Kettle Rock (KTL). Table 2 gives the position of the deployments, as well as the geographical size and number of devices. Table 3a–c summarizes the topographical features of the sensor station locations in each deployment. The number in the “Sensor station” column is the same as the one in Figure 7. “Slope” indicates the slope of the ground at the sensor station location. “Aspect” indicates the orientation of the slope relative to North. “Vegetation” indicates the percentage of vegetation at the sensor station location. This feature was estimated basing on the NLCD canopy dataset (https://www.mrlc.gov/). The original cell size of 30 m was downscaled to 10 m using bilinear interpolation for the scope of this work. Figure 7 shows a bird’s eye view of the deployments.

### 3.2. Examples of Hydrologic Data from WSNs

**Figure 8:**
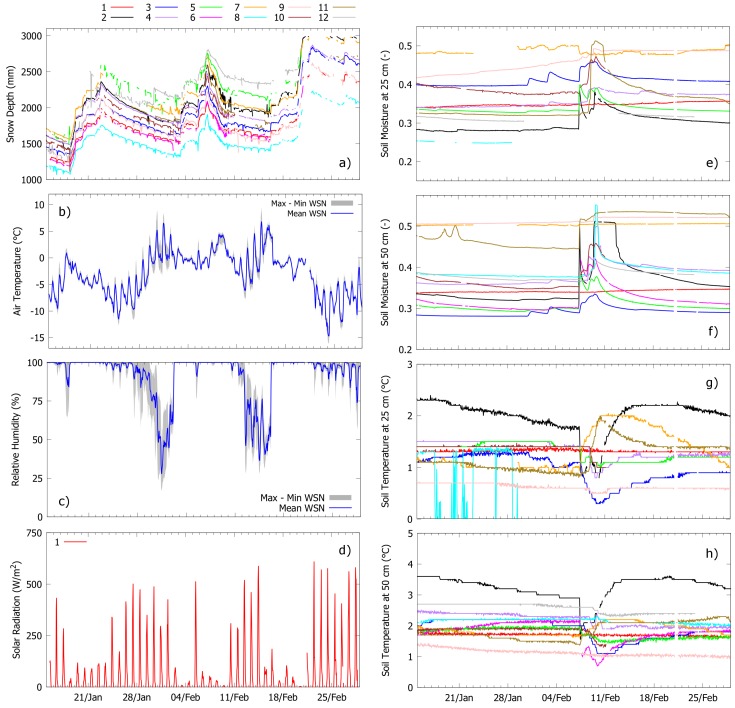
Examples of mid-winter sensor data from Grizzly Ridge (15 January 2017 to 1 March 2017). Line colors for panels (**e**–**f**) are the same as panel (**a**). Because measurements of air temperature and relative humidity show relatively small variability within nodes, panels (**b**,**c**) only report maximum-minimum range and mean. Solar radiation is only measured at node 1 (pillow) and shown in (**d**).

**Figure 9:**
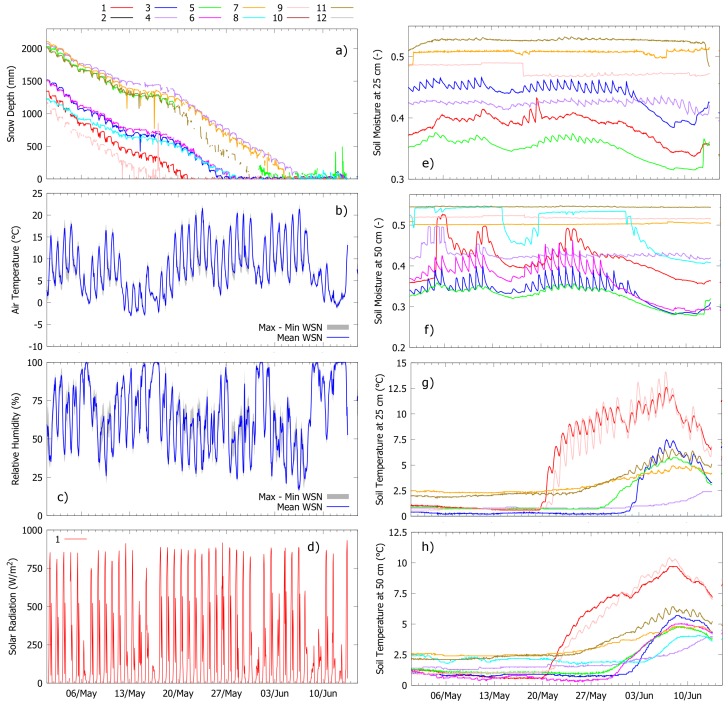
Examples of spring sensor data from Grizzly Ridge (1 May 2017 to 15 January 2017). Line colors for panels (**e**–**h**) are the same as panel (**a**). Because measurements of air temperature and relative humidity show relatively small variability within nodes, panels (**b**,**c**) only report maximum-minimum range and mean. Solar radiation is only measured at node 1 (pillow) and shown in (**d**). Soil moisture and temperature sensors at 25 cm depth malfunctioned at both nodes 6 and 8 during the reported periods. These data are therefore missing.

Figure 8 shows an example of mid-winter sensor data from Grizzly Ridge beginning 15 January 2017 and ending 1 March 2017. Figure 9 reports a second example from the same site, but in this case spans from 1 May 2017 to 15 Junuary 2017 (snowmelt season). These two temporal windows are used to exemplify the entire spectrum of hydrologic fluxes and states that were monitored during the 2016–2017 water year using wireless sensor networks.

#### 3.2.1. Accumulation Period

The 2016/2017 snow season at Grizzly Ridge started in mid-November but only a shallow snowpack persisted until 1 January (around 30 cm, data not shown). Between January and March, frequent atmospheric rivers from the Pacific Ocean hit the California coast and caused a marked increase in snow accumulation across the entire Sierra Nevada, making this water year one of the wettest on record (http://cw3e.ucsd.edu/how-many-atmospheric-rivers-have-hit-the-u-s-west-coast-during-the-remarkably-wet-water-year-2017/). In the Feather River, January and February were the wettest in 110 years of recorded data (Source: California Department of Water Resources (DWR)). Because of complex topographic transitions between rainfall and snowfall, some of these precipitation events exhibited both an increase in snow depth and massive snowmelt. An example is the rain-on-snow event between 6 and 10 February, when about 325 mm of precipitation fell over the basin (source: DWR), with dramatic consequences for the State’s water system and local population due to simultaneous damage of the Oroville dam spillway at the downstream outlet of the Feather River. Figure 8 focuses on three of these large precipitation events, which showed consistent patterns between nodes in terms of increasing snow depth (Figure 8a), saturated air (Figure 8c), and decreased solar radiation (Figure 8d). This similarity between nodes is due to the fact that precipitation events occur at a much larger scale than that of single sensor stations. Simultaneous measurements of snow depth and air temperature at nodes allow us to tentatively classify these events as either snowfall (18 January 2017 to 22 January 2017, and 19 February 2017 to 20 February 2017, average air temperature around −5.8/−0.6 ${}^{\circ }$C and −4.3/−0.1 ${}^{\circ }$C, respectively) or mixed rain and snow (3 February 2017 to 9 February 2017, average air temperature around −2.2/+4.4 ${}^{\circ }$C). Blending sensor information and co-located rain gauge data (https://cdec.water.ca.gov/) shows occurrence of rainfall after 8 February 2017, when snow depth at nodes started to decrease but the rain gauge recorded an increase. This pattern is again consistent with sensor data on relative humidity, radiation, and temperature (average air temperature around 0/+3 ${}^{\circ }$C). Relative humidity and temperature data show little variability between nodes during precipitation events and larger spatial heterogeneity during periods with no precipitation (e.g., 12 February 2017 to 15 February 2017, consistent with data of radiation and of the co-located rain gauge). These three precipitation events were separated either by periods of possible snowmelt (30 January 2017 to 31 January 2017, decreasing snow depth and temperatures above 0 ${}^{\circ }$C) or settling (25 January 2017 to 28 January 2017, again decreasing snow depth but temperatures below 0 ${}^{\circ }$C), with clearly different implications for runoff forecasting in snow-dominated contexts. Simultaneous soil moisture data (Figure 8e–f) show no significant infiltration during the two snowfall events but strikingly different patterns of soil moisture between nodes during the February rain-on-snow event. These increases and decreases in soil moisture may be related to differences in moisture conditions across nodes and in precipitation phase at local scale While nodes recorded stable winter soil temperature at seasonal scale (between +1 and +3 ${}^{\circ }$C), some of them showed either decreasing/increasing soil temperature from 7 February 2017 to 10 February 2017, which again could be related to local-scale energy processes during rain-on-snow events like snow pack phase change, soil thawing, or rainfall temperature.

#### 3.2.2. Snowmelt Period

The 2017 snowmelt season in Grizzly Ridge started in March. Depending on the location, canopy coverage, and peak snow depth of nodes, the end-of-season date ranged between 13 May 2017 (Node 9) and 6 June 2017 (Node 4). Figure 9 focuses on this key period of the water year when snowmelt runoff represents an important input to the surface and sub-surface hydrologic system of Californian Alpine watersheds.

All nodes showed a constantly decreasing snow depth during the period considered (Figure 9a). This decreasing trend due to snowmelt is consistent with simultaneous daily cycles in solar radiation (Figure 9d), relative humidity (Figure 9c), and temperature (Figure 9b), which are all proxies of stable atmospheric conditions and absence of precipitation (confirmed by the co-located rain gauge). The only period of constant snow depth was recorded between 14 May 2017 and 17 May 2017 and was marked by simultaneous negative air temperature, saturated air, and decreased solar radiation. While these conditions might be indicative of precipitation, a cross-check with snow depth data (constant) and co-located soil moisture data (decreasing at most nodes) can exclude significant precipitation events during this temporal window (again in agreement with the co-located rain gauge).

In terms of soil temperature, the end-of-season date was marked by diurnal temperature cycles that were not observed during periods of snow on the ground (Figure 9g–h, [38]). Shallower temperature probes (Figure 9g) showed more pronounced cycles than deeper sensors (Figure 9h), which is consistent with expected temperature profiles with depth. Soil moisture showed clear differences in daily temporal patterns between nodes (Figure 9e–f): while some nodes present recharge-discharge dynamics due to snowmelt infiltration into the ground, others show constant saturation, which may have impeded infiltration in favor of surface runoff. After snow disappeared, soil moisture decreased at most nodes due to the absence of inputs from the ground surface and concurrent evapotranspiration. The observed increase in superficial soil moisture at some nodes around 12 June 2017 to 15 June 2017 may be related to light rainfall and possibly snowfall on 12 June 2017 (minimum daily temperature around −1 ${}^{\circ }$C). This conclusion agrees with simultaneous relative humidity (Figure 9c) and radiation (Figure 9d) readings. In addition, some nodes measured a slight increase in snow depth, although on a scale comparable to background noise (see Figure 9a). The rain gauge did not measure any increase in precipitation, even though light rainfall/snowfall may be missed due to precision and under-catch.

#### 3.2.3. Comparison with Pre-Existing Survey Techniques: Snow Courses

**Figure 10:**
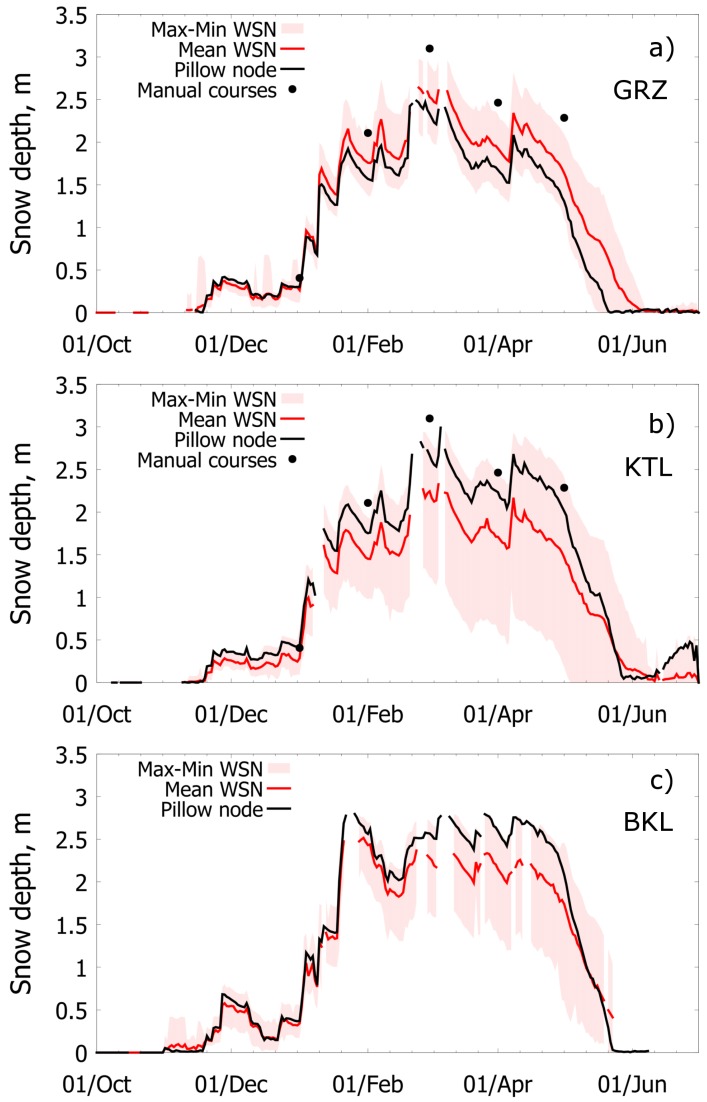
Comparison between snow depth measurements from the WSNs (red) with manual measurements taken by monthly snow courses (dotted black) at the same locations for Grizzly Ridge (**a**), Kettle Rock (**b**) and Bucks Lake (**c**) (no snow courses are done at the Bucks Lake site).

Figure 10 compares the range of variability of WSN snow depth measurements with manual measurements taken by monthly snow courses at the same locations (no snow courses are done at the Bucks Lake site). Snow courses are performed by manually measuring snow depth along transects and then averaging measurements to provide a representative value for the site. Daily snow depth at each node was estimated by calculating the median of all available readings on each day. Median values were preferred to means to reduce the impact of noise. Minimum, mean, and maximum snow depth across all nodes at a site were then calculated from these median values. These three statistics were calculated when at least eight different node values are available, which explains gaps in the time series. In addition, for the purposes of comparison, we highlight the depth recorded by the sensor node placed at the snow pillow. This node represents the same location as the pre-existing, standard snow and meteorological station. While this station also measures SWE, this variable is not directly measured by our wireless sensor networks.

The datasets show similar temporal patterns: accumulation occurs from December to February; peak accumulation in March; and snowmelt from April to May. Snow courses, however, tend to overestimate the mean site snow depth and may even exceed the maximum measurement from sensor nodes. The coarse temporal resolution of the snow courses makes it difficult to capture important hydrologic statistics such as date of peak snow or snow meltout date. The WSN data reveals that spatial variability increases over time in response to different solar radiation inputs across the nodes, mainly due to different aspects and vegetation coverage. This considerable variation cannot be captured by a single index station. Maximum differences in snow depth are on the order of 1.5–2 m, resulting in significantly different end-of-season dates from node to node: the difference between the first and last meltout date recorded by sensor nodes is 19 days in GRZ, 39 days (KTL), and 25 days (BKS). Since snowmelt is the primary driver of streamflow during the ablation period, this timing may significantly impact runoff forecasting. Snowfalls, on the other hand, reduce spatial variability since snow events are dictated by weather conditions at larger scales than that of WSNs. Several snow depth sensors saturated during last season, which means that the distance between the sensor and the surface of snow was too short for the sensor to make measurements. This was also treated as a node gap for the purposes of comparison.

### 3.3. Network Performance

#### 3.3.1. Estimated Performance

We use the Dust Networks SmartMesh Power and Performance Estimator (http://www.linear.com/docs/42452) [26] to calculate the performance of the network. Table 4 provides a full list of the input parameters. We use Figure 6a to count the number of devices at each hop. All other input parameters correspond exactly to the application deployed. Table 5 lists the key estimated performance indicators. The average current consumption of a device depends on its position in the network: the closer to the base station (the lower its “hop” value), the more stations it has to relay for, and the more current it consumes. All SmartMesh IP modules consume <50 μA. For repeater nodes that joined the network, that is the maximum current consumed (there are no other components). Since a repeater node is powered by a 17 Ah battery, this translates to tens of years of battery lifetime (A device consuming 49.7 μA should live for 39 years when powered by a 17 Ah battery. That being said, the shelf life of the Tadiran TLH-5930 D-cell battery is 20 years. The effective maximum lifetime is hence 20 years). A sensor station is equipped with many more electronics, including the PSOC and the sensors. The current draw of the SmartMesh IP module becomes negligible. The sensor station is powered by a 17 Ah battery pack, recharged by a 15 W solar panel, which is enough to perpetually power all electronics. Table 5 also indicates that the average latency (the time it takes for a sensor measurement to travel from the sensor station to the base station) is <6 s max. It takes <30 min for the entire network to build at installation. The network only builds once during the entire lifetime of the deployment; during that period, a device is “searching” for the network, consuming 500 μA on average.

#### 3.3.2. Measured Performance

Every 15 min, each mote generates a network statistic message that contains information about the mote itself and the neighbors it uses to communicate. Results in this section are extracted from over 7 million network statistics gathered from the three deployment sites.

When a mote transmits a packet, it waits for an acknowledge (ACK) to confirm that the receiver mote received the packet correctly. If the transmitting mote does not receive an ACK, it retransmits its packet. Because the motes are using channel hopping, retransmissions occur on a different channel than the first transmission, increasing the probability of reception [39]. The *Packet Delivery Ratio* (PDR) is the number of successful transmissions (i.e., transmissions that received an ACK) divided by the total number of transmissions. The PDR gives an idea of the “quality” of a wireless link.

Table 6 presents the measured PDR of the three sites over the following periods: (i) Bucks Lake from 23 September 2016 to 07 December 2016 (2.5 months—363,000 measurements); (ii) Grizzly Ridge from 24 September 2016 to 21 March 2017 (six months—1,209,000 measurements); and (iii) Kettle Rock from 9 October 2016 to 21 March 2017 (5.5 months—1,094,000 measurements).

**Figure 11:**
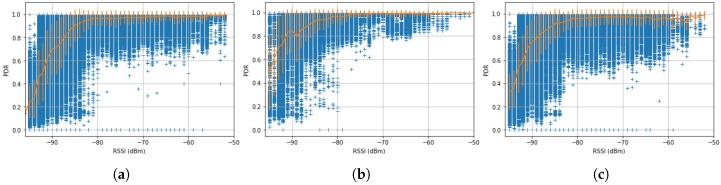
The PDR/RSSI “waterfall” plot: (**a**) Grizzly Ridge; (**b**) Bucks Lake; and (**c**) Kettle Rock.

To better understand the level of external interference, Figure 11 presents the relation between the RSSI and the PDR and shows the average and and standard deviation of the data in yellow. Those “waterfall plots” show that the average PDR of the links is very good (>95%) for transmissions above −80 dBm for every site. Below −80 dBm, the PDR decreases, indicating that frequent retransmissions is occurring on those links. They also indicate that the three sites do not suffer from external interferences, otherwise, the steep decrease plotted would be shifted to the right.

In SmartMesh networks, the deployment recommendation is that every node has to have at least 3 neighbors. To do so, each mote keeps track of the PDR of links to its neighbors, and periodically sends that information as regular data packets. This mechanism allows the software running on the base station to have a complete view of the connectivity in the network.

**Figure 12:**
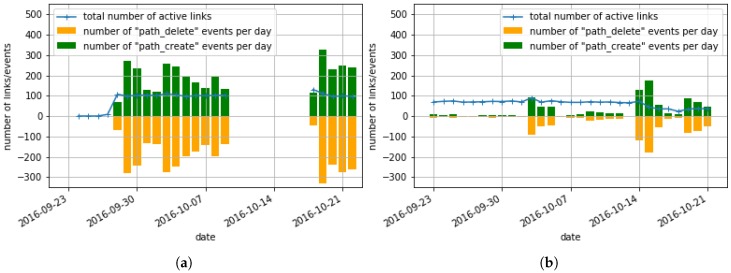
Network stability: the number of path_create and path_delete events generated per day over a month. The top line shows the total number of active links, as a reference: (**a**) Grizzly Ridge; and (**b**) Bucks Lake.

Every time a wireless link is created or deleted between neighbor nodes, these motes generate a path_create and path_delete event. We monitor those events to quantify the stability of the topology, or “churn”, which consumes energy. Figure 12 shows the number of path_create and path_delete events per day, over a month, for the Grizzly Ridge and Bucks Lake sites. The Kettle Rock site is not reported here as neighbors path_create and path_delete events were not collected at this site. The total number of links in the network is also depicted, as a reference.

At Bucks Lake, the churn alternates between periods with less than 10 events per day, and periods with almost 200 events per day. During the period with 10 events per day, once links are established, they remain useful for days/weeks at a time, resulting in a very stable topology. We attribute the high churn present in Grizzly Ridge to the lack of links with good quality (i.e., PDR > 70). As every mote tries to ensure it has at least two parents, it associates with neighbors even with a low quality link if no parent with high quality is present. Selecting low quality links highly increases the number of path_create and path_delete events. Installation of a couple more repeaters would solve this problem. At Grizzly Ridge, eight motes out of 45 (5.6%) generate 69% of the path events. Table 7 lists the motes with which sensor node 7 (i.e., the mote that generate the most events) communicated as well as the quality of the link with each of those motes and the number of network statistics gathered for each link (i.e., health reports). We can see that only two links have a PDR >30% and that one of these links was reported only twice, meaning that it was not available for the rest of the time. This means that most of the time, the sensor node 7 was looking for a second parent to associate with and had to select links with low quality. To solve that issue, the solution is to add one repeater mote to increase the density and thus, reduce the number of path events.

## 4. Discussion

WSNs provide dense spatio-temporal hydrologic data at physiographically representative locations. These data can support better real-time monitoring of hydrologic fluxes across the landscape (see Section 3.2). As demonstrated by [40], better hydrologic information can potentially increase hydropower revenue. To that end, this deployment of WSNs demonstrates the capability of collecting more comprehensive hydrologic data, which can potentially translate into lower uncertainty in streamflow forecasts at various temporal and spatial scales and improved economic viability of hydropower.

The design, deployment, and maintenance of wireless sensor networks require more effort and a higher budget compared to a standard weather station. The installation in high-mountain environments also poses challenges due to harsh, remote conditions; damage from wildlife; and potentially extreme weather conditions, as occurred during the 2017 water year. This section provides a broader context about existing snow hydrologic surveys to show the value of WSN hydrologic information and elaborates on strengths and challenges of this system during the extreme conditions of the past water year.

### 4.1. Value of the Hydrologic Product

WSNs provide several important advantages when compared with traditional index stations. Standard instrumentation often includes a snow pillow, rain gauge, temperature sensor, and possibly wind speed and radiation sensors. These instruments, especially snow pillows, are typically located in areas that are flat and free of vegetation, making them inherently biased estimates of snow distribution in alpine regions [41]. Snow pillows also prevent infiltration into the ground from the snow they are measuring and insulate snow from thermal exchanges with soil, further biasing data such as end-of-season date. The end-of-season date signals a shift from snowmelt-dominated runoff towards other processes like groundwater discharge and evapotranspiration, making it an important metric for hydropower forecasters.

WSNs can also compensate for some well-known problems with traditional sensors. For example, rain gauges provide information on precipitation amount, but not phase (rain or snow). They are also prone to under-catch during intense snowfall/rainfall events. Blending data from surface and subsurface sensors WSNs and co-located standard instrumentation allows us to detect precipitation timing and phase, which can be critical in determining the timing of subsequent streamflow peaks. Another example is infiltration: most existing networks do not routinely measure soil moisture, whereas our WSNs do. Since overland flow is a much faster process of streamflow generation than infiltration, soil moisture information can support short-term runoff forecasting at downstream reservoirs and powerhouses.

Finally, compared to traditional sites, WSNs can monitor how areas characterized by different canopy or aspect respond to precipitation, potentially allowing data collected under specific conditions to be generalized to uninstrumented areas with similar situations (see Section 4.5). Due to the complex interaction between snow melt and topography in mountain watersheds, data collected by traditional instruments are nearly impossible to distribute. WSNs, on the other hand, can tease out effects of canopy interception or geology on snowfall and snowmelt rate and infiltration patterns.

More specifically, snow depth and snow water equivalent are manually measured monthly using poles and vertical samplers, respectively [42]. Compared to WSN systems, manual surveys are more time consuming, sometimes risky in avalanche-prone areas, and only provide snapshots of snow accumulation patterns at specific sites at monthly or seasonal scales. They are often performed only in areas accessible during winter, such as flat, open areas where a helicopter can land.

Autonomous sensors have also been deployed on entire mountain ranges for both water resources monitoring and avalanche forecasting (https://www.wcc.nrcs.usda.gov/snow/, http://bcrfc.env.gov.bc.ca/data/asp/, http://www.jma.go.jp/jma/indexe.html, http://www.slf.ch, http://www.meteomont.gov.it/infoMeteo/mappaStazioniAutomatiche.do, https://www.nve.no/hydrology/, http://www.meteo.fr/temps/france/nivose/france_niv.html). Because of their often remote and distributed locations, data transmission within most of these networks is wireless, which make them technologically similar to WSNs. Compared to the latter, however, such systems lack spatial representativeness of their region because they are deployed as one index station per site [43]. Recent results by [44] for example show that traditional stations are not representative of actual mean SWE at a 1 km${}^{2}$ scale when compared to collocated WSNs. Moreover, traditional stations typically include only one of each type of sensor; should extreme alpine conditions damage the sensor midseason, the data are often lost. Finally, the footprint of autonomous sensors like snow pillows is significantly larger than that of single nodes. We estimate a minimum footprint for pillows in ∼10 m × 10 m including rain gauge and equipment shelter (https://www.wcc.nrcs.usda.gov/about/mon_automate.html). At a smaller scale, several examples exist of highly equipped snow stations whose extension match that of WSNs [45,46,47,48,49]. In a broader context, such observatories have for a long time represented the main source of data for experimental hydrology [50]. A commonly employed method for data transmission in intensive study plots consists of wiring peripheral sensors to a central manager or laboratory. These sensors can be more vulnerable in alpine conditions, as they do not feature the self-healing characteristics of WSNs. Experience from field deployments indicates, for example, that wires are frequently damaged by wildlife. In addition, wired systems are more invasive than wireless counterparts, which makes WSNs a preferred solution in remote locations.

Remote sensing represents the most recent innovation in snow surveys [19,20,21,22,23,24,25,42,51,52]. The spatial extent of remote sensing products is generally larger than WSNs, and sensors with different wavelengths allow them to capture a broad range of snow properties like albedo and snow wetness. On the other hand, the temporal resolution of available surveys is usually limited and hampered by cloud obstruction [25], whereas other techniques like laser scanning may be expensive and time consuming. Moreover they often require ground truthing from in-situ sensors or manual snow surveys. Some datasets, like MODIS, only provide direct information about snow covered area and are available at daily timesteps but at relatively coarse spatial scales (500 m), whereas other platforms only provide bi-weekly snapshots (see the US Landsat mission) or are still in an experimental phase (see for example the EU SENTINEL mission). Remote sensing is a promising complementary tool to WSNs, as it may provide information on broader spatial patterns that WSNs lack. WSNs provide the finer temporal resolution necessary for short-term streamflow forecasting. In California, for example, the Airborne Snow Observatory is now providing maps of snow depth at an unprecedented spatial resolution [21], even though the spatial and temporal extent of these scans is still limited by budgetary constraints. Synergy between these techniques can potentially provide the necessary data needed by water resources managers in real time.

### 4.2. Design Choices: Comparison with Other Wireless Solutions

Numerous wireless solutions—both academic and off-the-shelf commercial—are available which may be considered for our application. Below, we offer a brief comparison and justification for selecting the SmartMesh IP system.

Low-power wide-area network (LPWAN) technology has received attention in the last year, with two competing approaches, Sigfox and LoRA. They are similar in that compliant radios send small frames to one or more base stations up to 15 km away. The range makes it a very appealing technology, and remote environmental monitoring could be an ideal target application. However, we have identified several potential drawbacks which rule out LPWAN for our purposes.

First, the amount of data LPWAN technology can carry is too little. A Sigfox node, for example, can send only 140 frames per day, each carrying only 8 B of payload. This is roughly an order of magnitude below what our sensor stations produce. Second, though both Sigfox and LoRA offer some downstream capability (the ability to send commands to the device), it is not comprehensive enough for our use. Only a handful of frames per day can be sent; this is several orders of magnitude too little and prevents the user from being able update firmware remotely. Over-the-air reprogramming, especially of the firmware, is a crucial requirement, as our deployments are inaccessible during the entire winter. Third, LPWAN technologies are "best-effort"; that is, when a device sends a frame, it has no way of knowing whether a base station received it. For example, early field trials of LoRA show end-to-end reliability as low as 90%, even with thirteen base stations ([in French] http://www.orange-business.com/fr/blogs/usages-dentreprise/machine-to-machine/qualite-de-service-d-un-reseau-iot-base-sur-lorawantm-enseignements-et-elements-mis-en-oeuvre). For our application, it is critical to lose as little data as possible, as real-time forecasting of the yield of hydroelectric power plants are based on the data collected. Finally, proven technology that offers wire-like reliability already exists, which can be seamlessly integrated with a single cheap cellular uplink connection. These more viable options, coupled with the fact that no LPWAN technologies have been deployed in the Feather River basin, make LPWAN technology a less than ideal option.

In terms of the base station’s connection to the Internet in remote regions, a few options exist depending on availability. Cellular connection is the most attractive in terms of data rate and pricing. Cellular coverage can be limited in remote areas, but directional antennas can improve connections. Otherwise satellite linkage must be used. This is not preferred as, compared to cellular, satellite costs far more to transmit and is finicky to maintain.

#### Rationale for using SmartMesh IP

The analysis above has lead us to opt for SmartMesh IP, coupled with a single cellular connection at the base station. The result is a complete end-to-end solution with key benefits, which we list below.

**Low complexity and cost.** The low-power wireless network connects all sensor stations to the base station locally at the deployment site. This means that only the base station (not the sensor stations) needs to connect to the Internet, improving reliability and resiliency of the system.

**Multi-km${}^{2}$ deployment area.** Given its multi-hop nature, a sensor station can be arbitrarily far from the base station. A deployment can span several km${}^{2}$.

**Ultra low-power operation.** The SmartMesh IP modules consume <50 μA on average, allowing over a decade of battery lifetime.

**Wire-like reliability.** SmartMesh IP was designed for critical industrial applications, and offers over 99.999% end-to-end reliability.

**Fully bi-directional communication.** At any point in time, a network administrator or monitoring program can send commands to any of the devices in the network. This ability permits, for example, tuning parameters midseason.

**Over-the-air reprogramming.** As a corollary to having bi-directional communication, all SmartMesh IP modules can be securely reprogrammed over-the-air.

**Built-in diagnostics.** Each SmartMesh IP device regularly generates diagnostic data allowing a network administrator to have full visibility over the health of the network.

**Proven and truly off-the-shelf.** Over 60,000 SmartMesh networks have been deployed so far. One vendor alone, Emerson, claims over 31,900 networks, with cumulated node operating hours above 9 billion (http://www.emerson.com/en-us/expertise/automation/industrial-internet-things/pervasive-sensing-solutions/wireless-technology). While SmartMesh IP was designed for industrial applications, it has been used in numerous other spaces, including smart buildings (http://versasense.com/), smart cities (http://www.linear.com/docs/41387) and smart agriculture [53]. SmartMesh IP is a proven technology; we chose to use it because our system operates well within the limits of a SmartMesh IP network. For example, SmartMesh IP network as a whole cannot generate more than 36 packets per second, with each packet carrying at most 90 bytes of application payload. In our case, each sensor station generates a 20-byte data packet every 15 min, well within the limit. The typical latency from a sensor station to the base station is on the order of 1–2 s. Given the relatively slow-moving nature of the data that we are measuring, this delay is acceptable.

### 4.3. Comparison with Existing WSN Systems for Snow Monitoring

Multiple research projects, described below, aim to use WSNs to monitor snow properties. A 57-node WSN was successfully deployed across a forested, 1-km${}^{2}$ headwater catchment in the southern Sierra Nevada of California using SmartMesh IP technology as the system backbone but with different sensors, hardware and software design [34]. The software and hardware used did not allow for data recovery. It was determined that a 50-m node-to-node spacing would conservatively lead to a good PDR. More importantly, [34] highlight the importance of network reconfiguration during the actual deployment using information of RSSI and PDR collected by the network to avoid network collapse. A histogram of PDR values showed that after readjustment, about 80% of all network paths are performed “within the desired 85-90% design value, and over 50% of all paths are at 100% PDR” [34]. On the other hand, we deployed repeaters at distances greater than 50 m whenever line-of-sight between them was available. Thanks to the newly developed SOL, we were able to visualize in real-time the quality of links in terms of RSSI and PDR, detect issues, and identify potential adjustments (see Figure 7). Moreover, the base station in [34] is installed at the network edge, which not only represents a regional point of failure, but also increases the operational burden and power consumption of repeaters close to the base station given that they must route all incoming network packets to the base station.

A similar system of 14 WSNs was deployed in the high elevations of the American River basin to measure the snowpack in real-time [44]. Each WSN consists of 10 sensor stations placed within a 1 km${}^{2}$ area. Zhang et al. [44] frame the system as capable of long-term operation with minimal maintenance and highlight the ease of installation. The system uses the same networking hardware as our deployments, which is capable of multi-level storage. However, it uses different sensors and has no data-recovery functionality. Moreover it uses different deployment strategies for base stations, sensor stations, and repeaters as the one described in Section 2.4, as well as a different software suite. The paper also calls for future work to develop tools to verify the performance of the network, interfaces to assist during network deployment, visualization of the network health information, real-time displays of sensor data, and logging of maintenance activity, all of which are implemented by the system presented in this paper.

Henderson et al. [54] plan to build 50–100 WSNs to monitor and forecast avalanches in the Wasatch Mountains in Utah, by measuring different properties of the snow. Their mote will use a chipcom CC1000 RF transceiver by Texas instruments that will need interfacing and considerable low-level protocol design and programming to reach an efficient and usable wireless system adequate for alpine environments. The research work presented is still in its starting phase where only lab tests of sensors have been performed with no indications of field deployment.

SnowFort [55] presents a full WSN-based system for infrastructure and environmental monitoring with server-side data analytics. Although the main focus of their work is structural health monitoring, the framework described is meant to fit broader applications such as snow monitoring. The system’s high-level conceptual design is similar to the one presented in this article. The main system components are the TelosB, used as mote, and a Raspberry-pi, used as a base station. TelosB is an 18-year-old technology developed as a teaching tool at the University of California Berkeley. It is programmed via the older tinyOS, another teaching tool. The device uses an 8-bit MCU and is hindered by one megabyte of on-board storage memory. Unlike the system presented in this paper, SnowFort only supports single-hop star network topology, which presents a spatial coverage issue. SnowFort’s suggestion for increasing spatial coverage is to install multiple base stations. However, base stations can consume orders of magnitude more power than motes and even more when they transmit data to the Internet. Each base station would require a cell network connection. This represents a shortcoming for monitoring snow across spacious alpine regions.

Conceptually, the closest WSN-based system found in the literature is SnowCloud [56], which uses the TelosB with different core mesh protocol and components. This system has been deployed at the Sagehen Creek, CA experimental field station [57]. Nodes communicate via a TinyOS network. The core component of each sensor station is a MEMSIC TelosB mote, with all the issues described above. Their sensor station consists of two parts: a surface node that is very similar to our sensor node, and a ground node that communicates wirelessly with the surface node through the snow. A critical difference between the two systems is power consumption at the sensor node: while the TelosB platform has a 20 to 30 mA consumption on average, a NeoMote consumes 2 mA on average. Authors rightly note that “network time synchronization would certainly provide a more robust system and allow nodes to periodically operate in low-power mode”. This is at the core of our system provided by the SmartMesh IP mesh protocol and for SnowCloud, this can be achieved by jettisoning the use of tinyOS and using openWSN [58]. They are also currently developing the gateway capability of the manager and remote time synchronization of the sensor station’s real-time clock to combat clock drifts. Both of those features are present in our system.

### 4.4. Challenges and Lessons Learned

The key variable for choosing among the most appropriate technological solutions available on the market was the ability to hold up under the harsh alpine environment like of the California Sierra Nevada. In-lab and local field testing further the range of potential failures found in the field. Still, several problems ranging from minor to critical were encountered during the system operation. The following lessons learned would improve future deployments:

1. We experienced prolonged power failures at the Bucks Lake manager due to the misplacement of the manager node in a poorly irradiated location shaded by canopy. The manager cell modem was configured to shutdown at 11 V to stop draining the battery, which was devoted to powering the WSN network manager. This allowed the WSN to keep operating locally, but without real-time publishing, a major issue for a real-time system. Both Kettle and Grizzly managers were better placed and did not exhibit such a problem, highlighting the need for considering canopy coverage during the design phase. Additional batteries were added to the Bucks Lake base station to prevent future outages. Relocating the solar panel could also be a solution, where/when feasible.
2. Some repeaters disconnected due to the original design of repeater layout. A choice was initially made to connect the repeater antennas through the top of the repeater box, sealing the mechanical connection with silicone caulk to prevent water seepage into the enclosure. Poor construction of the antennas prevented water from draining out of the bottom of the vented antenna. This disconnected some nodes from the network. Real-time link health maps Figure 7 allowed for the timely discovery of the issue, and after drilling small holes in the clogged antennas, repeaters became functional again.
3. A firmware/hardware bug prevented some sensor nodes from sampling and sending data after a power recovery from a total battery discharge. The bug was attributed to the gradual voltage increase during recharge that mainly affected the real time clock component. The problem was subsequently fixed by adding a power-up voltage threshold and a delay to guarantee the different NeoMote components are operational before the main code starts. Only a few nodes exhibited this behavior, which was resolved by the code update.
4. We experienced extensive rodent damage to exposed antenna, sensor and solar power wires, especially at Bucks Lake. The cables close to the ground were all in metal conduit but the wires from the solar panel and temp/rH at the 5 m level were exposed. The 5 m of snow in 2017 allowed the pesky rodents to access these exposed wires. System resiliency can be improved by appropriately shielding all wires from wildlife.
5. Solar panels, antennas, and snow depth sensors at several nodes were buried in snow for a few days during peak accumulation. This design issue was due to the abundant precipitation that occurred in the 2017 winter (we estimate about 4000 mm of total precipitation at Bucks Lake, with peak SWE around 1400 mm). This season demonstrated that choosing the most suitable height *a priori* depends on consideration of extremes and could be difficult in a context of climate change-related extreme weather events. We recommend allowing for unanticipated extreme events during the design phase. In particular, efforts should be made to keep the base and sensor stations’ antennas and the solar panel at the base station functional, as this is the most sensitive part of the network, Sensor stations can last several months on a full charge, so buried solar panels were of limited consequence. In addition, redundancy of nodes at the same site makes the network resilient to localized failures compared to standard index stations.

The potential impacts of these problems was limited by the network’s multi-level data replication feature, which means that data are stored at the sensor node, manager node, and server-side allowing for multi-layer data recovery. When disconnected sensor nodes rejoin the network, they automatically resend previously unsent data, safely stored on the internal SD card, allowing for a more timely recovery.

In view of the above problems, we can identify a few best practices for future deployments:

1. **Hierarchy of criticalities**: In such large-scale systems, it is important to identify and classify system elements based on their importance to the overall system operation. For instance, the base station power and connectivity to the Internet are far more critical than that of a sensor node, which in turn is more important than that of a repeater.
2. **Adequate Testing**: In-lab testing for such systems is crucial. Moreover, testing in similar but easily accessible environments would also be an asset. Failures of temperature and humidity sensors that were then observed in the field first occurred in the UC Botanical Garden test network where weather conditions are closer to the mountains than lab settings.

### 4.5. Future R&D Directions

This work opens up numerous research directions, both from a systems and a hydrologic perspective. While WSNs represent a well-established alternative to traditional sensing systems in many applications (see above), their use as a decision-support system for hydropower is relatively new and several improvements could be put in place to streamline their use in operational hydrology.

From a networking point of view, we are currently testing new server capabilities to provide advanced real-time network health analysis. This is key information from a decision support standpoint. We currently have the ability to visualize the quality of network links, giving administrators an intuitive interface to analyze network health. We are working to generate notifications of certain events, such as a downed link or an extreme snow melt/accumulation. Such improvements are needed to make these systems more user-friendly and expand their use outside academic or experimental case studies. We are also working on improving the sensor stations’ firmware to reduce the join duty cycle in the event that they lose connectivity to the network. Finally, work is being done to allow remote reprogramming of the PSOC on the sensor stations to increase flexibility and seriously reduce cost of field operations.

From a network planning point of view, we are working on tools to help a network installer with positioning repeater and sensor nodes in tandem. This tool builds on previous work on placing sensor stations [30] based on propagation models in alpine environments [36]. The result will be a tool which, given environmental information about the deployment site, identifies the optimal repeater locations to ensure good connectivity within the network. Maintaining, moving, or replacing repeaters has represented an important part of our summer fieldwork after the first winter of operations. This emphasizes repeaters as a crucial component of a WSN that has received little attention in terms of deployment strategies compared to sensor stations. Replacing trial-and-error techniques for repeater placement with more automatic (and repeatable) techniques could increase the applicability of WSNs in real-world applications. However, this would require better pre-characterization of canopy properties, e.g., LIDAR, compared to available satellite-based images, which could increase the overall cost of deployment. More research is needed to determine whether it is worth pursuing. Future studies could also explore optimization methods for the overall system design to ensure long-term operation at minimal cost (explored in the context of WSNs monitoring oil pipelines by [59]) and assessing overall system reliability (e.g., through a Markov-model of the system evolving in multiple environments [60]).

Finally, we intend to generate real-time SWE maps by blending our WSN data with remote sensed products such as MODIS and Landsat fractional snow cover. These spatial snowpack maps can then be assimilated into runoff models such as PRMS in an attempt to improve reservoir inflow forecasting. From a hydrologic perspective, this is the most important direction of future developments and the real testing ground for the value of WSNs. Snow patterns are highly variable in space and time and this heterogeneity has important feedbacks with various aspects of the biosphere, including vegetation distribution and streamflow timing during the dry season. While these results show potential for an improved monitoring of hydrologic fluxes at locations that are representative of relevant physiographic features, leveraging this information to provide real-time and spatially consistent information at catchment scale will expand the dataset and provide more useful tools for water resources managers. A specific challenge here is to conceive multi-cluster WSNs that can expand monitoring capabilities of single networks along large altitudinal, longitudinal, and latitudinal ranges that could better meet the typical scale of interest of hydrology.

## Figures and Tables

**Table 1:** Fields contained in a SOL object.

Field symbol	Field content
**M**	address of the device which created the object
**T**	timestamp of when the object was created
**t**	type of the object, as defined in the SOL registry
**L**	length of the value field
**V**	value of the object

**Table 2:** Location and size of deployments.

	Bucks Lake	Grizzly Ridge	Kettle Rock
Latitude	39.850000	39.917000	40.140000
Longitude	−121.242000	−120.645000	−120.715000
Deployment area	20 ha	27 ha	42 ha
Num. sensor stations	12	12	12
Num. repeater nodes	22	25	31
Num. base stations	1	1	1
Total num. devices	**35**	**38**	**44**

**Table 3:** Sensor station features.

**(a) Bucks Lake**				
**Sensor Station**	**Elevation (m asl)**	**Slope (${}^{\circ }$)**	**Aspect (${}^{\circ }$)**	**Vegetation (%)**
0	1752.18	4.87	237.70	69
1	1739.24	12.73	272.04	65
2	1769.00	0.45	158.07	52
3	1715.75	14.70	276.87	69
4	1768.86	2.15	109.49	66
5	1754.57	9.60	318.66	57
6	1702.77	17.03	221.94	84
7	1771.00	2.00	132.53	70
8	1753.43	4.45	89.80	24
9	1736.49	7.69	323.98	43
10	1700.23	14.04	338.58	77
11	1744.54	3.84	53.72	71
Mean (site)	1746.35	8.39	198.33	65
25${}^{\circ }$ perc.	1737.28	4.35	92.49	60
75${}^{\circ }$ perc.	1758.34	11.74	314.79	77
**(b) Grizzly Ridge**				
**Sensor Station**	**Elevation (m asl)**	**Slope (${}^{\circ }$)**	**Aspect (${}^{\circ }$)**	**Vegetation (%)**
1	2083.36	4.68	15.25	13
2	2063.50	11.09	53.60	70
3	2101.94	5.18	102.18	55
4	1997.44	15.23	57.83	63
5	2098.09	17.77	348.08	67
6	2109.13	10.49	327.77	55
7	2075.89	6.10	109.41	38
8	2081.81	3.24	73.01	19
9	2019.66	11.09	47.71	51
10	2115.61	7.15	324.20	51
11	2015.63	12.17	59.33	41
12	2070.13	16.44	39.50	73
Mean (site)	2089.93	9.30	131.73	48
25${}^{\circ }$ perc.	2075.19	5.35	39.76	34
75${}^{\circ }$ perc.	2113.57	12.05	228.65	64
**(c) Kettle Rock**				
**Sensor Station**	**Elevation (m asl)**	**Slope (${}^{\circ }$)**	**Aspect (${}^{\circ }$)**	**Vegetation (%)**
1	2228.09	17.96	196.39	45
2	2239.26	7.80	231.00	40
3	2276.69	12.30	153.34	45
4	2171.84	14.96	179.93	88
5	2198.68	13.51	54.50	35
6	2166.72	14.18	154.13	58
7	2210.55	8.20	179.41	0
8	2234.77	14.89	98.82	2
9	2217.44	11.40	213.00	50
10	2157.93	8.99	156.45	61
11	2131.82	15.29	179.94	63
12	2234.41	11.67	13.83	32
Mean (site)	2213.69	10.64	159.40	42
25${}^{\circ }$ perc.	2180.32	8.25	142.26	23
75${}^{\circ }$ perc.	2246.90	12.95	174.52	61

**Table 4:** Performance Estimator input.

**Hop**	**Number of Devices**
1	5
2	8
3	9
4	9
5	2
6	1
**Input Parameter**	**Value**
Requested service	900 s
Reporting interval	900 s
Payload size	50 B
Hardware type	5800 8 dBm
Supply voltage	3.6 V
Downstream frame size	1024
Join duty cycle	10%

**Table 5:** Performance Estimator output.

**Hop**	**Average Current**	**Mean latency**
1	49.7 μA	0.95 s
2	38.7 μA	1.87 s
3	37.3 μA	2.79 s
4	29.5 μA	3.70 s
5	32.2 μA	4.62 s
6	27.2 μA	5.54 s
**Estimated Performance Indicator**		**Value**
Manager ave. current		218 μA
Network build time		24.1 min
Mote search current		500 μA

**Table 6:** Measured average PDR over the three deployment sites.

	Bucks Lake	Grizzly Ridge	Kettle Rock
**Average PDR**	89%	79%	82%
**PDR stand. dev.**	16%	22%	20%
(Transmit/Fails)	(15,654 K/1757 K)	(64,027 K/13,297 K)	(15,654 K/1757 K)

**Table 7:** List of neighbors of sensor node 7 at Grizzly Ridge, with link quality and number of associated health reports. The mote does not constantly have two neighbors with high PDR that it can use as parents. It thus has to associate with neighbors with low link quality. Repeater nodes 03, b7, f9, 4c, ac are annotated in Figure 7c.

Destination Node	PDR	#HR
Repeater node d3	14%	349
Repeater node 03	11%	72
Repeater node b7	9%	4
Repeater node f9	97%	1422
Repeater node ad	100%	2
Repeater node 4c	14%	73
Repeater node ac	9%	22
Sensor node 8	25%	1297

## References

[1] Romanov P., Gutman G., Csiszar I. (2000). Automated Monitoring of Snow Cover over North America with Multispectral Satellite Data. J. Appl. Meteorol..

[2] Barnett T., Adam J., Lettenmaier D. (2005). Potential Impacts of a Warming Climate on Water Availability in Snow-dominated Regions. Nature.

[3] Sturm M., Goldstein M.A. (2017). Water and Life from Snow: A Trillion Dollar Science Question. Water Resour. Res..

[4] Laghari J. (2013). Climate Change: Melting Glaciers Bring Energy Uncertainty. Nature.

[5] Rheinheimer D.E., Viers J.H., Sieber J., Kiparsky M., Mehta V.K., Ligare S.T. (2014). Simulating High-Elevation Hydropower with Regional Climate Warming in the West Slope, Sierra Nevada. J. Water Resour. Plan. Manag..

[6] Ho M., Lall U., Allaire M., Devineni N., Kwon H.H., Pal I., Raff D., Wegner D. (2017). The Future Role of Dams in the United States of America. Water Resour. Res..

[7] Finger D., Heinrich G., Gobiet A., Bauder A. (2012). Projections of Future Water Resources and Their Uncertainty in a Glacierized Catchment in the Swiss Alps and the Subsequent Effects on Hydropower Production during the 21st Century. Water Resour. Res..

[8] Zhang Z., Glaser S.D., Watteyne T., Malek S. (2016). Long-term Monitoring of the Sierra Nevada Snowpack Using Wireless Sensor Networks. IEEE Internet Things J..

[9] Milly P., Betancourt J., Falkenmark M., Hirsch R.M., Zbigniew W., Lettenmaier D.P., Stouffer R.J. (2008). Stationarity Is Dead: Whither Water Management?. Science.

[10] Markstrom S.L., Regan R.S., Hay L.E., Viger R.J., Webb R.M., Payn R.A., LaFontaine J.H. (2015). PRMS-IV, the Precipitation-Runoff Modeling System, Version 4.

[11] Lehning M., Völksch I., Gustafsson D., Nguyen T.A., Stähli M., Zappa M. (2006). ALPINE3D: A Detailed Model of Mountain Surface Processes and its Application to Snow Hydrology. Hydrol. Process..

[12] Martinec J., Rango A., Roberts R., Baumgartner M.F. (1998). Snowmelt Runoff Model (SRM) User’s Manual.

[13] Bavera D., Bavay M., Jonas T., Lehning M., De Michele C. (2014). A Comparison Between Two Statistical and a Physically-based Model in Snow Water Equivalent Mapping. Adv. Water Resour..

[14] Elder K., Cline D., Liston G.E., Armstrong R. (2009). NASA Cold Land Processes Experiment (CLPX 2002/03): Field Measurements of Snowpack Properties and Soil Moisture. J. Hydrometeorol..

[15] Serreze M.C., Clark M.P., Armstrong R.L., McGinnis D.A., Pulwarty R.S. (1999). Characteristics of the Western United States Snowpack from Snowpack Telemetry (SNOTEL) Data. Water Resour. Res..

[16] Ryan W.A., Doesken N.J., Fassnacht S.R. (2008). Evaluation of Ultrasonic Snow Depth Sensors for U.S. Snow Measurements. J. Atmos. Ocean. Technol..

[17] Avanzi F., De Michele C., Ghezzi A., Jommi C., Pepe M. (2014). A Processing-Modeling Routine to use SNOTEL Hourly Data in Snowpack Dynamic Models. Adv. Water Resour..

[18] Johnson J.B., Gelvin A.B., Duvoy P., Schaefer G.L., Poole G., Horton G.D. (2015). Performance Characteristics of a New Electronic Snow Water Equivalent Sensor in Different Climates. Hydrol. Process..

[19] Prokop A., Schirmer M., Rub M., Lehning M., Stocker M. (2008). A comparison of measurement methods: Terrestrial laser scanning, tachymetry and snow probing for the determination of the spatial snow-depth distribution on slopes. Ann. Glaciol..

[20] Revuelto J., Vionnet V., López-Moreno J.I., Lafaysse M., Morin S. (2016). Combining Snowpack Modeling and Terrestrial Laser Scanner Observations Improves the Simulation of Small Scale Snow Dynamics. J. Hydrol..

[21] Painter T.H., Berisford D.F., Boardman J.W., Bormann K.J., Deems J.S., Gehrke F., Hedrick A., Joyce M., Laidlaw R., Marks D. (2016). The Airborne Snow Observatory: Fusion of Scanning Lidar, Imaging Spectrometer, and Physically-based Modeling for Mapping Snow Water Equivalent and Snow Albedo. Remote Sens. Environ..

[22] Bühler Y., Adams M.S., Bösch R., Stoffel A. (2016). Mapping Snow Depth in Alpine Terrain with Unmanned Aerial Systems (UASs): Potential and Limitations. Cryosphere.

[23] De Michele C., Avanzi F., Passoni D., Barzaghi R., Pinto L., Dosso P., Ghezzi A., Gianatti R., Vedova G.D. (2016). Using a Fixed-wing UAS to Map Snow Depth Distribution: An Evaluation at Peak Accumulation. Cryosphere.

[24] Harder P., Schirmer M., Pomeroy J., Helgason W. (2016). Accuracy of Snow Depth Estimation in Mountain and Prairie Environments by an Unmanned Aerial Vehicle. Cryosphere.

[25] Dietz A.J., Kuenzer C., Gessner U., Dech S. (2012). Remote Sensing of Snow—A Review of Available Methods. Int. J. Remote Sens..

[26] Watteyne T., Weiss J., Doherty L., Simon J. Industrial IEEE802.15.4e Networks: Performance and Trade-offs. Proceedings of the IEEE International Conference on Communications (IEEE ICC).

[27] Pister K.S.J., Doherty L. TMSP: Time Synchronized Mesh Protocol. Proceedings of the IASTED International Symposium Distributed Sensor Networks.

[28] Brun-Laguna K., Watteyne T., Malek S., Zhang Z., Oroza C., Glaser S.D., Kerkez B. SOL: An End-to-end Solution for Real-world Remote Monitoring Systems. Proceedings of the 2016 IEEE 27th Annual International Symposium on Personal, Indoor, and Mobile Radio Communications (PIMRC).

[29] Bray T. (2014). The JavaScript Object Notation (JSON) Data Interchange Format.

[30] Oroza C.A., Zheng Z., Glaser S.D., Tuia D., Bales R.C. (2016). Optimizing Embedded Sensor Network Design for Catchment-scale Snow-depth Estimation Using LiDAR and Machine Learning. Water Resour. Res..

[31] Lee S., Younis M. (2012). Optimized relay node placement for connecting disjoint wireless sensor networks. Comput. Netw..

[32] Mehajabin N., Razzaque M.A., Hassan M.M., Almogren A., Alamri A. (2016). Energy-sustainable relay node deployment in wireless sensor networks. Comput. Netw..

[33] Kashyap A., Khuller S., Shayman M. Relay placement for higher order connectivity in wireless sensor networks. Proceedings of the 25th IEEE International Conference on Computer Communications.

[34] Kerkez B., Glaser S.D., Bales R.C., Meadows M.W. (2012). Design and Performance of a Wireless Sensor Network for Catchment-scale Snow and Soil Moisture Measurements. Water Resour. Res..

[35] Rice R., Bales R.C. (2010). Embedded-sensor network design for snow cover measurements around snow pillow and snow course sites in the Sierra Nevada of California. Water Resour. Res..

[36] Oroza C.A., Zhang Z., Watteyne T., Glaser S.D. (2017). A Machine-Learning Based Connectivity Model for Complex Terrain Large-Scale Low-Power Wireless Deployments. IEEE Trans. Cognit. Commun. Netw..

[37] SmartMesh IP Application Notes. http://cds.linear.com/docs/en/application-note/SmartMesh_IP_Application_Notes.pdf.

[38] Lundquist J.D., Lott F. (2008). Using Inexpensive Temperature Sensors to Monitor the Duration and Heterogeneity of Snow-covered Areas. Water Resour. Res..

[39] Watteyne T., Mehta A., Pister K. Reliability Through Frequency Diversity: Why Channel Hopping Makes Sense. Proceedings of the International Symposium on Performance Evaluation of Wireless Ad Hoc, Sensor, and Ubiquitous Networks (PE-WASUN).

[40] Rheinheimer D.E., Bales R.C., Oroza C.A., Lund J.R., Viers J.H. (2016). Valuing year-to-go hydrologic forecast improvements for a peaking hydropower system in the Sierra Nevada. Water Resour. Res..

[41] Zhang Z., Glaser S.D., Bales R.C., Martha C., Robert R., Daniel M. (2017). Insights into Mountain Precipitation and Snowpack from a Basin-scale Wireless Sensor Network. Water Resour. Res..

[42] Sturm M. (2015). White Water: Fifty Years of Snow Research in WRR and the Outlook for the Future. Water Resour. Res..

[43] Bales R.C., Molotch N.P., Painter T.H., Dettinger M.D., Rice R., Dozier J. (2006). Mountain Hydrology of the Western United States. Water Resour. Res..

[44] Zhang Z., Glaser S.D., Bales R.C., Martha C., Daniel M. (2017). Technical Report: The Design and Evaluation of a Basin-scale Wireless Sensor Network for Mountain Hydrology. Water Resour. Res..

[45] Leppänen L., Kontu A., Hannula H.R., Sjöblom H., Pulliainen J. (2016). Sodankylä Manual Snow Survey Program. Geosci. Instrum. Methods Data Syst..

[46] Reba M.L., Marks D., Seyfried M., Winstral A., Kumar M., Flerchinger G. (2011). A Long-term Data Set for Hydrologic Modeling in a Snow-Dominated Mountain Catchment. Water Resour. Res..

[47] Krajči P., Kirnbauer R., Parajka J., Schöber J., Blöschl G. (2017). The Kühtai Data Set: 25 Years of Lysimetric, Snow Pillow, and Meteorological Measurements. Water Resour. Res..

[48] Wever N., Schmid L., Heilig A., Eisen O., Fierz C., Lehning M. (2015). Verification of the Multi-layer SNOWPACK Model with Different Water Transport Schemes. Cryosphere.

[49] Morin S., Lejeune Y., Lesaffre B., Panel J.M., Poncet D., David P., Sudul M. (2012). An 18-yr Long (1993–2011) Snow and Meteorological Dataset from a Mid-altitude Mountain Site (Col de Porte, France, 1325 m alt.) for Driving and Evaluating Snowpack Models. Earth Syst. Sci. Data.

[50] Blöschl G., Blaschke A.P., Broer M., Bucher C., Carr G., Chen X., Eder A., Exner-Kittridge M., Farnleitner A., Flores-Orozco A. (2016). The Hydrological Open Air Laboratory (HOAL) in Petzenkirchen: A Hypothesis-driven Observatory. Hydrol. Earth Syst. Sci..

[51] Parajka J., Blöschl G. (2006). Validation of MODIS Snow Cover Images over Austria. Hydrol. Earth Syst. Sci..

[52] Margulis S.A., Cortés G., Girotto M., Durand M. (2016). A Landsat-Era Sierra Nevada Snow Reanalysis (1985–2015). J. Hydrometeorol..

[53] Watteyne T., Diedrichs A.L., Brun-Laguna K., Chaar J.E., Dujovne D., Taffernaberry J.C., Mercado G. (2016). PEACH: Predicting Frost Events in Peach Orchards Using IoT Technology. EAI Endorsed Trans. Int. Thing.

[54] Henderson T., Grant E., Luthy K., Cintron J. Snow Monitoring with Sensor Networks. Proceedings of the 29th Annual IEEE International Conference on Local Computer Networks.

[55] Liao Y., Mollineaux M., Hsu R., Bartlett R., Singla A., Raja A., Bajwa R., Rajagopal R. (2014). SnowFort: An Open Source Wireless Sensor Network for Data Analytics in Infrastructure and Environmental Monitoring. IEEE Sens. J..

[56] Skalka C., Frolik J. Snowcloud: A Complete Data Gathering System for Snow Hydrology Research. Proceedings of the 5th International Workshop, REALWSN 2013.

[57] Moeser C.D., Walker M., Skalka C., Frolik J. Application of a Wireless Sensor Network for Distributed Snow Water Equivalence Estimation. Proceedings of the Annual Western Snow Conference.

[58] Watteyne T., Vilajosana X., Kerkez B., Chraim F., Weekly K., Wang Q., Glaser S., Pister K. (2012). OpenWSN: A standards-based low-power wireless development environment. Trans. Emerg. Telecommun. Technol..

[59] Xia C., Liu W., Deng Q. (2015). Cost minimization of wireless sensor networks with unlimited-lifetime energy for monitoring oil pipelines. IEEE/CAA J. Autom. Sin..

[60] Liu B., Cui L., Si S., Wen Y. (2016). Performance measures for systems under multiple environments. IEEE/CAA J. Autom. Sin..

